# An Accurate and Computationally Efficient Model for Membrane-Type Circular-Symmetric Micro-Hotplates

**DOI:** 10.3390/s140407374

**Published:** 2014-04-23

**Authors:** Usman Khan, Christian Falconi

**Affiliations:** Department of Electronic Engineering, University of Tor Vergata, Via del Politecnico 1, 00133 Rome, Italy; E-Mail: Usman.Khan@uniroma2.it

**Keywords:** circular-symmetric micro-hotplates, temperature distribution, gas sensors, infrared emitters, micro-reactors

## Abstract

Ideally, the design of high-performance micro-hotplates would require a large number of simulations because of the existence of many important design parameters as well as the possibly crucial effects of both spread and drift. However, the computational cost of FEM simulations, which are the only available tool for accurately predicting the temperature in micro-hotplates, is very high. As a result, micro-hotplate designers generally have no effective simulation-tools for the optimization. In order to circumvent these issues, here, we propose a model for practical circular-symmetric micro-hot-plates which takes advantage of modified Bessel functions, computationally efficient matrix-approach for considering the relevant boundary conditions, Taylor linearization for modeling the Joule heating and radiation losses, and external-region-segmentation strategy in order to accurately take into account radiation losses in the entire micro-hotplate. The proposed model is almost as accurate as FEM simulations and two to three orders of magnitude more computationally efficient (e.g., 45 s *versus* more than 8 h). The residual errors, which are mainly associated to the undesired heating in the electrical contacts, are small (e.g., few degrees Celsius for an 800 °C operating temperature) and, for important analyses, almost constant. Therefore, we also introduce a computationally-easy single-FEM-compensation strategy in order to reduce the residual errors to about 1 °C. As illustrative examples of the power of our approach, we report the systematic investigation of a spread in the membrane thermal conductivity and of combined variations of both ambient and bulk temperatures. Our model enables a much faster characterization of micro-hotplates and, thus, a much more effective optimization prior to fabrication.

## 1. Introduction

Micro-hotplates comprise an integrated resistive heater within a thin membrane [1]. The membrane is thermally isolated from the substrate by bulk etching in order to achieve a low power consumption [2] and to ensure safety to other devices (e.g., electronic interface [3], sensors/actuators and possibly, in future, energy harvesters [4–6]) operating on the same chip [7]. As to the applications, micro-hotplates are widely utilized in gas sensors [8–10], infrared emitters [11,12] and micro-reactors [7,13]. Nevertheless, the available simulation tools for micro-hotplates are not yet satisfactory. In fact, when designing a certain device, it is generally necessary to carefully take into account both spread (*i.e.*, statistical variations of relevant parameters) and drift (*i.e.*, aging and variations of the operating conditions). For instance, electronic circuits must always be designed by taking into account the so called PVT variations (*i.e.*, the variations of process parameters, voltage supply, and temperature). By contrast, at present, a similar approach is practically impossible for micro-hotplates. Though both the operating temperature and the uniformity of the temperature profile within the heater region are crucial [14,15], the computational cost of FEM simulations (currently the only available tool allowing an accurate simulation of micro-hotplates) is very high. For this reason, a systematic and effective analysis of micro-hotplates is still an open challenge and, generally, it is impossible to accurately investigate the effects of all the many important variables, which include:

– the thermal conductivity and emissivity of the materials constituting a micro-hotplate;
– the electrical resistivity of the heater at a reference temperature;
– the temperature coefficient of the heater resistivity;
– the geometry and dimensions of the micro-heater (including widths of the tracks, separations between the adjacent tracks and thickness);
– the geometry and dimensions of the membrane (radius/width and thickness);
– boundary conditions (*i.e.*, the convection heat transfer coefficient, the bulk temperature and ambient temperature).

As an alternative to FEM simulations, analytical models could be employed. Though several efforts have been carried out to model the thermal behavior in membrane-type micro-hotplates [16–23], the available models are clearly insufficient. For example, Kozlov [16,17] modeled the temperature distribution in micro-hotplates using the Fourier method. However, in [16], the radiation heat transfer was linearized by assuming a small temperature difference between the micro-hotplate and the environment, which is generally not the case for micro-hotplates. In order to circumvent this concern, in [17], Kozlov, for each region of interest, considered a modified environmental temperature defined as the mean of the typical environmental temperature and the average region temperature; however, obviously, in the heater-regions of typical micro-hotplates the difference between the average region temperature and the modified environmental temperature is still large. Moreover, the heater geometries in [16,17] are also hypothetical. Volklein *et al.* [18] and Giberti *et al.* [19] considered a constant temperature inside the heater and, therefore, their expressions may give no information on the temperature uniformity. Li *et al.* developed analytical expressions in terms of Bessel functions [20]; however, first, they applied these relations to a rectangular micro-hotplate structure (thus introducing large errors) and, second, they also linearized the radiation heat transfer assuming a small temperature difference between the sensor and the environment. Jain *et al.* [21] have developed a model for the temperature distribution in membrane-type micro-hotplates with a line heat source, but neglected both convection and radiation heat transfers and, unlike the most widely used structures, utilized a line heat source. Graf *et al.* [22] also ignored the radiation heat transfer and focused on FEM simulations without providing an analytical solution.

Recently, we have shown [23] that, for an hypothetic membrane-type micro-hot-plate with perfectly circular geometry (e.g., made of several concentric circular regions) and sufficiently small thickness, the temperature distribution in each circular region can be expressed by a linear combination of modified Bessel functions with the coefficients to be determined using boundary conditions. Subsequently, we have introduced simple strategies for designing practical (*i.e.*, not perfectly circular) membrane-type micro-hotplates with an almost-circular temperature distribution [24], which will be referred to as circular-symmetric micro-hotplates. However, the modified Bessel functions expression for the temperature developed in [23] has two major shortcomings. Firstly, it does not consider the internal heat generation within the heater regions; in fact, accurately modelling the internal heat generation is not straightforward as it would require taking into account the resistivity of the heaters and its temperature dependence. Secondly, the radiation heat transfer is approximated by means of the Taylor first-order polynomial centered at the average temperature of the region of interest and, therefore, the predicted temperature is only accurate if there are no large temperature differences within the region of interest. Since the temperature external to the heater region can be very different from its average temperature, the approximation of the radiation losses would not be accurate in the external region. We stress that neglecting the radiation outside the heater would have resulted in a constant error of the temperature distribution in the heater region which can be, e.g., larger than 10 °C; such error is unimportant for estimating the temperature uniformity in the heater region (as it is a “constant error”) but does affect the average temperature of the heater. In order to overcome these shortcomings, here, we first develop a general expression for the temperature distribution in terms of modified Bessel functions which also takes into account the Joule heating within the micro-heater, where the temperature dependence is linearized using the Taylor first order polynomial. Afterwards, we introduce an external-region-segmentation approach which divides the external region into many small concentric annular regions with small maximum temperature differences, so that the temperature dependence of the radiation heat flow can be accurately linearized within each annular region; as a result, the radiation heat transfer can be taken into account in the entire device. Clearly, the annular regions must be more dense and small in the proximity of the heater region, where high temperature gradients exist, and less dense close to the substrate. However, as a consequence of the external-region-segmentation, the micro-hotplate comprises a large number of annular regions and, therefore, a large number of unknown coefficients of modified Bessel functions to be determined in order to find the specific expression for the temperature in each annular region. For these reasons, we also introduce a matrix-based approach that automatically forms a set of linear equations, using the relevant boundary conditions, whose solution vector gives the coefficients of modified Bessel functions in each concentric annular region. Conventional matrix-based software for numerical calculations can be utilized to effectively perform matrix operations. Additionally, since our approach requires the average temperatures of each annular region in the linearization process, we have adopted an iterative approach, similar to [17], to calculate the average temperatures. In case of multiple analyses, similar to circuit simulators (e.g., SPICE), our model takes advantage of the average temperatures found in the previous analysis as initial guesses, thus further improving the computational efficiency of our approach.

In conclusion, our model enables the accurate and computationally efficient analysis of circular-symmetric membrane-type micro-hotplates by taking advantage of modified Bessel functions, of an external-region-segmentation, and of a matrix approach for determining the relevant boundary conditions. Our model is complete as, unlike any previous model [16,17,20,21], takes into account the internal heat generation and its temperature dependence, and all the three heat transfer mechanisms, *i.e.*, conduction, convection and radiation. The paper is organized as follows. First, considering the circular structure and small thickness, we discuss the general solution including the internal heat generation and the heat transfers by the conduction, convection and radiation. Afterwards, we present our external-region-segmentation approach which allows to accurately model the radiation heat transfer in the complete device. Thereafter, we present our matrix-based approach for finding the specific solutions in each region of a three-heater micro-hotplate. Later, we introduce the iterative process and the utilization of results from previous simulations as initial guesses for the case of multiple simulations. As an illustrative example, we have applied our model to predict the temperature distribution in a three-heater micro-hotplate and also analyzed its performance in terms of a spread in the thermal conductivity of the membrane and of a combined variation of ambient and bulk temperatures; we also compared the results with FEM simulations in terms of accuracy and computational efficiency. Finally, in order to reduce the small and constant errors due to the undesired heating in the electrical contacts, we introduce a single-FEM-compensation strategy.

## 2. Modeling of the Temperature Distribution in Circular-Symmetric Micro-Hotplates

**Figure 1.**
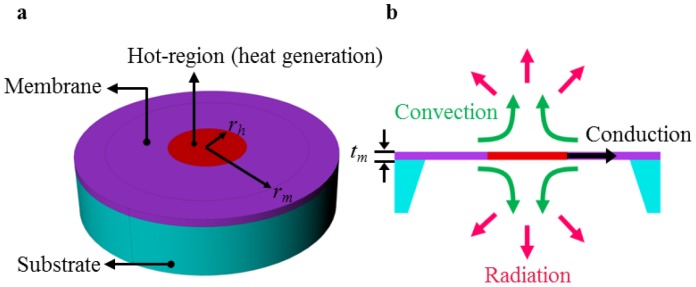
Schematic diagram for a circular-symmetric micro-hotplate and the corresponding heat flows. Perspective view of the micro-hot-plate structure showing different parts (**a**); Cross-sectional view of the micro-hotplate structure with the description of the three heat transfer mechanisms (**b**).

Let us consider the micro-hotplate schematically shown in Figure 1, where *t_m_* is the thickness of the membrane, *r_m_* is the radius of the membrane, and *r_h_* is the radius of the hot region (*i.e.*, the area whose temperature must be high and as close as possible to the desired one). In general, there is a heat generation in the hot-region, conduction heat transfer through the membrane, and convection and radiation heat transfers at both the top and the bottom surfaces of the membrane. For a perfectly circular structure with typically very small thickness, as shown in Figure 1, the temperature in the entire membrane only depends on the distance from the center, *r* [23].

**Figure 2.**
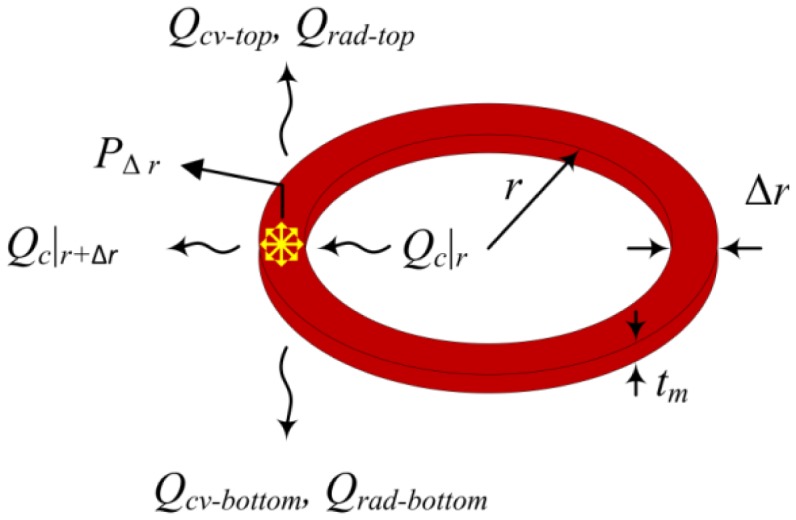
Heat flows for a thin cylindrical ring.

As schematically shown in Figure 2, if we consider a thin cylindrical ring within the membrane and apply the thermal energy balance we find:

(1) $${Q_{c}|}_{r+\Delta r}-{Q_{c}|}_{r}-P_{\Delta r}+Q_{cv-top}+Q_{cv-bottom}+Q_{rad-top}+Q_{rad-bottom}=0$$

where *P*_Δ_*_r_* is the heat generated in the cylindrical ring, *Q_c_* is the heat flow due to conduction, *Q_cv_*_-_*_top_* and *Q_cv_*_-_*_bottom_* are the convection heat flows from the top and bottom surfaces, *Q_rad_*_-_*_top_* and *Q_rad_*_-_*_bottom_* are the radiation heat flows from the top and bottom surfaces. Importantly, unlike [23], we have also incorporated the internal heat generation while applying the thermal energy balance to the cylindrical ring:

(2) $$P_{\Delta r}=p\left(2\pi r\Delta r\right)t_{m}$$

(3) $$Q_{c}=q_{c}\left(2\pi rt_{m}\right)$$

(4) $$Q_{cv,\text{top}}+Q_{cv,\text{bottom}}=2h_{c}\left(2\pi r\Delta r\right)\left(T-T_{a}\right)$$

(5) $$Q_{\text{rad},\text{top}}+Q_{\text{rad},\text{bottom}}=2\sigma \varepsilon \left(2\pi r\Delta r\right)\left(T^{4}-T_{a}^{4}\right)$$

where *p* is the volumetric density of the internal heat generation (obviously *p* is zero in the regions which do not include heaters), *σ* is Stefan's Boltzmann constant, *q_c_* is the heat flux for the conduction in the radial direction, *2πr*Δ*rt_m_* is the volume of the thin cylindrical ring, *2πrt_m_* is the cross sectional area for the conduction, *2πr*Δ*r* is the surface area for the convection and radiation, *T_a_* is the ambient temperature, *ε* is the average surface emissivity of the membrane and *h_c_* is the average convection heat transfer coefficient (average refers to the fact that the emissivities and convection heat transfer coefficients can be different at the top and bottom surfaces). The convection heat transfer coefficient *h_c_* is difficult to determine as it depends on different parameters (geometry, packaging, environment, … [25]); however, we mention that it must be determined prior to using our method by means of FEM simulations and/or experiments [26,27].

The internal heat generation in micro-hotplates is due to Joule heating within the resistive heating elements. However, due to the temperature dependence of the heater resistance, the internal heat generation is also a function of the temperature. In order to consider such temperature dependence, we may approximate the heat generated in a part of a resistive heater, *P*, as:

(6) $$P≃\frac{V^{2}}{R_{T\_\mathrm{avg}}\left[1+\alpha \left(T-T_{avg}\right)\right]}$$

where *R_T_avg_* is the resistance at the average temperature, *T_avg_* is the average temperature within the region under consideration, *α* is the temperature coefficient of the heater resistivity at the average temperature, and *V* is the voltage across the resistor. The temperature dependence of the internal heat generation can be linearized using the Taylor first order polynomial:

(7) $$P≃\frac{V^{2}}{R_{T\_avg}}\left[1-\alpha \left(T-T_{avg}\right)\right]$$

Importantly, instead of using the resistance at ambient temperature in Expressions (6) and (7), we have utilized the resistance at the average temperature *R_Tavg_* as *T*−*T_avg_* is much less than *T*−*T_a_* and, therefore, the Taylor approximation holds well. As a result, the volumetric power density *p* = *P*/(*S_R_t_m_*), where *S_R_* is the surface area of the region under consideration, can be written as:

(8) $$p=P_{0}-P_{1}\left(T-T_{avg}\right)$$

$$p_{0}=\frac{V^{2}}{R_{T\_\mathrm{avg}}\left(S_{R}t_{m}\right)}\;\text{and}\;p_{1}=\frac{\alpha V^{2}}{R_{T\_\mathrm{avg}}\left(S_{R}t_{m}\right)}$$

Though the resistive heating elements constitute only a small part of the membrane thickness, for simplicity, we assume that the internal heat generation is taking place along the entire thickness while computing the volumetric power density; this simplification is not critical as the membrane thickness in the typical micro-hotplates is extremely small. Combining Equations (2) and (8), we get the expression for the internal heat generated within the thin cylindrical ring:

(9) $$P_{\Delta r}=\left[p_{0}-p_{1}\left(T-T_{\mathrm{avg}}\right)\right]\left(2\pi r\Delta r\right)t_{m}$$

As to the radiation heat loss, by taking advantage of the first-order Taylor series expansion centered at *T* = *T_avg_*, we can linearize the Expression (5) [23]:

(10) $$Q_{\text{rad}-\text{top}}+Q_{\text{rad}-\text{bottom}}=2\sigma \varepsilon \left(2\pi r\Delta r\right)\left(4T_{\mathrm{avg}}^{3}\right)\left[T-\left(\frac{3T_{\mathrm{avg}}}{4}+\frac{T_{a}^{4}}{4T_{\mathrm{avg}}^{3}}\right)\right]$$

If we now substitute (3), (4), (9) and (10) into the expression for the thermal energy balance (1), we find:

(11) $$\begin{array}{r} {q_{c}(2\pi rt_{m})|}_{r+\Delta r}-{q_{c}(2\pi rt_{m})|}_{r}-\left[p_{0}-p_{1}\left(T-T_{avg}\right)\right]\left(2\pi r\Delta r\right)t_{m}+2h_{c}(2\pi r\Delta r)\left[T-T_{a}\right]\\ +2\sigma \varepsilon \left(2\pi r\Delta r\right)\left(4T_{avg}^{3}\right)\left[T-\left(\frac{3T_{avg}}{4}+\frac{T_{a}^{4}}{4T_{avg}^{3}}\right)\right]=0 \end{array}$$

Substituting *q_c_*= −*k*[*dT*(*r*)/*dr*] (where *k* is the thermal conductivity of the membrane) and simplifying (11) as in [23].

(12) $$\frac{d^{2}\left(T\right)}{dr^{2}}+\frac{1}{r}\frac{d\left(T\right)}{dr}-\frac{\left(2h_{c}+8\sigma \varepsilon T_{\mathrm{avg}}^{3}+p_{1}t_{m}\right)}{kt_{m}}\left[T-\frac{\left(2h_{c}T_{a}+6\sigma \varepsilon T_{\mathrm{avg}}^{4}+2\sigma \varepsilon T_{a}^{4}+p_{0}t_{m}+p_{1}t_{m}T_{\mathrm{avg}}\right)}{\left(2h_{c}+8\sigma \varepsilon T_{\mathrm{avg}}^{3}+p_{1}t_{m}\right)}\right]=0$$

This is a modified (the third term is negative rather than positive) Bessel differential equation of *zero-th* order and has a general solution [23,28]:

(13) $$T\left(r\right)=C_{1}I_{0}\left(n_{g}r\right)+C_{2}K_{0}\left(n_{g}r\right)+T_{g}$$

where:

$$\begin{array}{c} T_{g}=\left(2h_{c}T_{a}+6\sigma \varepsilon T_{\mathrm{avg}}^{4}+2\sigma \varepsilon T_{a}^{4}+p_{0}t_{m}+p_{1}t_{m}T_{\mathrm{avg}}\right)/\left(2h_{c}+8\sigma \varepsilon T_{avg}^{3}+p_{1}t_{m}\right)\\ n_{g}=\sqrt{\left(2h_{c}+8\sigma \varepsilon T_{\mathrm{avg}}^{3}+P_{1}t_{m}\right)/kt_{m}} \end{array}$$

*C*_1_ and *C*_2_ are the constants that must be determined by applying boundary conditions, *I_i_* = a modified Bessel function of the 1st kind and *i*-*th* order where [*dI*_0_(*n_g_r*)/*dr*]=*n_g_I*_1_(*n_g_r*)[23] and *K_i_* = a modified Bessel function of the 2nd kind and *i*-*th* order where [*dK*_0_(*n_g_r*)/*dr*]= −*n_g_K*_1_(*n_g_r*)[23].

This expression for the temperature distribution in membrane-type circular-symmetric micro-hot-plates, compared with [23], also considers the internal heat generation and its temperature dependence (due to the temperature coefficient of resistances); clearly, for regions without internal heat generation, the terms *p_0_* and *p_1_* are zero. Remarkably, apart from the conduction and convection, Equation (13) also includes the radiation heat transfer, under the assumption that the radiation heat transfer can be accurately described by the first order Taylor polynomial centered at the average temperature of the region under consideration. Therefore, in a certain annular region of the micro-hotplate, Equation (13) is very accurate if and only if the temperature within that region is sufficiently close to the average temperature of the annular region. This is not an issue for the annular regions inside the hot-region (where *T*−*T_avg_* must be low for ensuring good temperature uniformity within the hot-region), but may be difficult in the external region where the temperature ranges from very high values (in the proximity of the hot-region) to the environment temperature (in the proximity of the bulk).

### External-region-segmentation strategy

Here, unlike [23], we introduce an external-region-segmentation strategy for considering the radiation heat transfer in the external region as well. The strategy is about dividing the external region into a number of annular regions such that *T*−*T_avg_* is reasonably small within each annular region and, consequently, the Taylor approximation is accurate. Obviously, the annular regions must be more dense (and small) close to the hot-region, where the temperature rapidly falls from the high hot-region temperature to much lower temperatures; however, since the temperature gradients in the proximity of the bulk are much smaller, the annular regions can be less dense close to the bulk.

**Figure 3.**
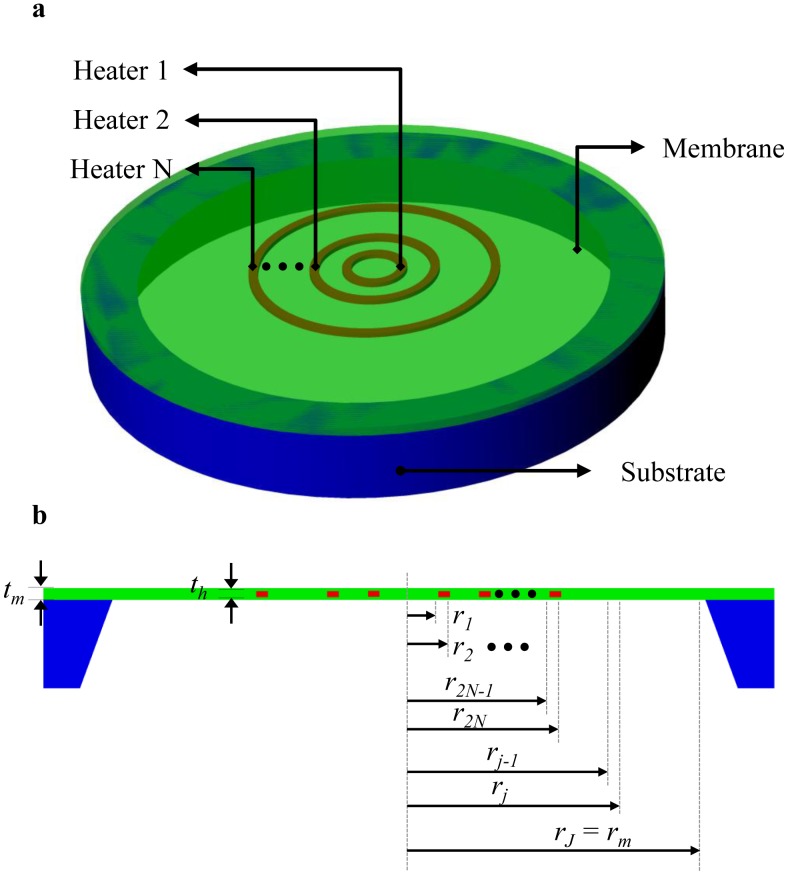
Perspective (**a**) and cross-sectional (**b**) views of a circular-symmetric micro-hotplate with N ring-shaped-heaters.

The micro-hot-plate structure shown in Figure 1 has only two regions: a central hot-region and an external-region. Such a structure can be practically realized as a circular micro-hotplate with a single heater producing an almost-constant power per unit area. However, this approach typically results in poor temperature homogeneity because of the very different conduction heat flows at the center (small temperature gradients across the membrane) and at the periphery (high temperature gradients across the membrane) of the hot region. In order to achieve a better temperature uniformity within the hot-region, some works [26,29,30] have utilized two heaters where the inner heater and the peripheral heater separately provide the different heat flows required to achieve an almost-constant temperature within the hot region. In practice, in [23], it has been observed that maximizing the temperature uniformity within the hot-region requires to keep both the inner heater and the peripheral heater at the same temperature as this choice corresponds to null, in the region comprised between the inner and outer heaters, the average linear dependence of temperature on the distance from the center; as a result, the temperature distribution will have two (nominally identical) maxima in correspondence of the inner and outer heaters, respectively. Clearly, the minimum temperature within the hot region will be found between the inner and outer heaters and, according to the analytical relations found in [23], the minimum temperature will be reduced by decreasing the distance between the inner and outer heaters. It is, therefore, obvious that a multi-heater design with more than two heaters within the hot-region can further improve the temperature uniformity by reducing the length of the “passive” regions between the adjacent heaters. Therefore, in order to formulate our matrix-based approach for finding the unknown coefficients of modified Bessel functions (in Equation (13)) in different micro-hotplate regions, let us consider a general circular micro-hot-plate with N ring-shaped-heaters as shown in Figure 3. With N heaters, the total number of regions J is 2N + *N_s_*_-_*_ext_*_-_*_region_* where *N_s_*_-_*_ext_*_-_*_region_* is the external-region-segmentation factor and it should be chosen so high that the Taylor's approximation holds well external to the hot region. Therefore, Equation (13) can be re-written for *j*-*th* region of the micro-hot-plate shown in Figure 3 as:

(14) $$T_{j}\left(r\right)=C_{1,j}I_{0}\left(n_{g,j}r\right)+C_{2,j}K_{0}\left(n_{g,j}r\right)+T_{g,j},\;j=1,2,...,\text{J}$$

(15) $$T_{g,j}=\frac{\left(2h_{c}T_{a}+6\sigma \varepsilon T_{\mathrm{avg},j}^{4}+2\sigma \varepsilon T_{a}^{4}+p_{0,j}t_{m}+p_{1,j}t_{m}T_{\mathrm{avg},j}\right)}{\left(2h_{c}+8\sigma \varepsilon T_{\mathrm{avg},j}^{3}+p_{1,j}t_{m}\right)}$$

(16) $$n_{g,j}=\sqrt{\frac{\left(2h_{c}+8\sigma \varepsilon T_{\mathrm{avg}}^{3}+p_{1,j}t_{m}\right)}{k_{j}t_{m}}}$$

where *T_avg_*_,_*_j_* is the average temperature of *j*-*th* region and *k_j_* is the thermal conductivity of *j*-*th* region. In the parts of the micro-hotplate which are made of a stack of materials (e.g., metallic heaters integrated on top of the membrane or within two different layers), the thermal conductivity of region *j* is given by [16]:

(17) $$\begin{array}{c} k_{j}=\frac{\overset{n}{\underset{i=1}{\text{∑}}}a_{i,j}t_{i,j}k_{i,j}}{t_{e,j}}\\ t_{e,j}=\overset{n}{\underset{i=1}{\text{∑}}}a_{i,j}t_{i,j} \end{array}$$

where *a_i,j_* is the ratio of the area of layer *i* in region *j* to the total area of the region, *t_i,j_* and *k_i,j_* are the thickness and thermal conductivity, respectively, of layer *i* in region *j* and *t_e,j_* is the effective thickness of region *j*.

Since the internal heat generation is present only within the ring-shaped-heaters, for all the even values of *j*, which correspond to the regions within the hot-region, *j*=2, 4, 6,…, 2*N*:

(18) $$\begin{array}{c} p_{0,j}=\frac{{V_{i}}^{2}}{R_{i,T\_avg}\pi \left[{r_{j}}^{2}-r_{j-1}^{2}\right]t_{m}}\\ p_{1,j}=\frac{{\alpha V_{i}}^{2}}{R_{i,T\_avg}\pi \left[{r_{j}}^{2}-r_{j-1}^{2}\right]t_{m}} \end{array}$$

where *i*=*j*/2 and *V_i_* is the voltage across the *i-th* heater which has the resistance *R_i_*_,_*_Tavg_* at the average temperature of the region.

The internal heat generation is zero in all the other regions or, equivalently, for all the odd values of *j* inside the hot region and for all the values of *j* which correspond to the regions external to the hot region, *i.e.*, for *j*=1, 3, 5,…,2*N* −1, 2*N*+1, 2*N*+2,…, *J*:

(19) $$p_{0,j}=0\;\text{and}\;p_{1,j}=0$$

The coefficients of modified Bessel functions, which are the unknown, can be determined using the boundary conditions which are:

– the temperature continuity at the interfaces between the adjacent regions;
– the heat flow continuity at the interfaces between the adjacent regions;
– no heat flow at the center of the micro-hotplate;
– the bulk temperature *T_b_* at the edge of the membrane.

By applying the condition of no heat flow at the center to Equation (14), we find:

(20) $$C_{1,\text{I}}I_{1}\left(0\right)-C_{2,\text{I}}K_{1}\left(0\right)=0$$

Starting from Equation (14), by applying the temperature and heat flux continuity to *jth* interface between *jth* and (*j*+*1*)*th* region, we find:

(21) $$\begin{array}{r} C_{1,j}I_{0}\left(n_{g,j}r_{j}\right)+C_{2,j}K_{0}\left(n_{g,j}r_{j}\right)-C_{1,j+1}I_{0}\left(n_{g,j+1}r_{j}\right)-C_{2,j+1}K_{0}\left(n_{g,j+1}r_{j}\right)=T_{g,j+1}-T_{g,j},\\ j=1,2,3,\ldots ,J-1 \end{array}$$

(22) $$\begin{array}{r} k_{j}n_{g,j}C_{1,j}I_{1}\left(n_{g,j}r_{j}\right)-k_{j}n_{g,j}C_{2,j}K_{1}\left(n_{g,j}r_{j}\right)-k_{j+1}n_{g,j+1}C_{1,j+1}I_{1}\left(n_{g,j+1}r_{j}\right)+k_{j+1}n_{g,j+1}C_{2,j+1}K_{1}\left(n_{g,j+1}r_{j}\right)\\ =0,\;j=1,2,3,\ldots ,J-1 \end{array}$$

By applying the condition of the bulk temperature at the membrane edge to (14), we have:

(23) $$C_{1,J}I_{0}\left(n_{g,J}r_{J}\right)+C_{2,J}K_{0}\left(n_{g,J}r_{J}\right)=T_{b}-T_{g,J},r_{J}=r_{m}$$

### Matrix-based approach

The analytical solutions for the unknown coefficients in Equations (20)–(23) are very complex, but all the coefficients can be easily determined by taking advantage of a numerical matrix-based approach. In fact, we can re-write the set of Equations(20)–(23) as a single matrix equation:

(24) $$BC=T_{c}$$

where **B** is a square 2J × 2J matrix, **C** is a 2J × 1 matrix (column vector) whose elements are the unknown coefficients of modified Bessel functions and **T_c_** is a 2J × 1 matrix (column vector) whose elements are the terms on the right hand sides of Equations (20)–(23). The solution vector, whose elements are the unknown coefficients, to Equation (24) is given by:

(25) $$C=B^{-1}T_{c}$$

The matrix Equation (25) can be easily solved by conventional tools for numerical computing (e.g., MATLAB, Scilab, …). In conclusion, after finding the coefficients by solving Equation (25), the Equation (14) can be applied to all the annular regions and thus provide the temperature distribution throughout the micro-hot-plate.

### Iterative process and initial guesses

In order to linearize the radiation heat transfer (10), our model requires the average temperatures of all the annular regions, *T_avg_*_,_*_j_*, as input data. Therefore, similar to [17], we utilize an iterative process in order to find the average temperatures of the regions. Initially, we set the ambient temperature as the average temperature for each region and then, by using the iterative process, we compute the temperature distributions in all the regions and use these intermediate temperature distributions for computing the average temperatures to be used in the next step. The algorithm converges when the error between the former and the current value of the average temperature becomes smaller than a desired threshold (though, of course, the number of iterations depend on this threshold, reducing the threshold to small values, e.g., below 1 °C, does not improve the accuracy as a small error in the estimation of the Taylor center does not significantly affect accuracy). Moreover, similar to circuit simulators (such as SPICE), when running multiple simulations, we have used the results found in the previous simulation as initial guesses for the current simulation, thus further improving the computational efficiency. We also mention that, in the absence of an accurate experimental data, we have not included, in our model, the temperature dependence of the thermal conductivity and convection coefficients [17]. However, our model can consider such temperature dependences by using the same iterative approach as in [17]; in practice, while executing an iteration, the iterative approach has to modify the value of the temperature dependent parameters according to the average temperature of the region found in the previous step.

## 3. Comparison with FEM Simulations

Here, we compare our model with FEM simulations for the validation and for verifying its superior computational efficiency. The computational efficiency is especially crucial for simulating micro-hot-plates as, first, many design parameters can significantly affect the temperature distribution and, second, a multitude of simulations are generally necessary for designing a practical device.

We have carried out FEM simulations in COMSOL utilizing the Joule heating module and triangular sweep meshing; FEM simulation considers the heat transfer by all three mechanisms, *i.e.*, conduction, convection and radiation. As to the materials, the micro-hot-plate consists of silicon nitride membrane and platinum heater; the material properties are summarized in Table 1. Since the thermal conductivities for the thin films of silicon nitride and platinum are much less than the bulk values, here, we have utilized the values reported in [31,32] for the thin films. Unless otherwise specified, we assumed the bulk temperature equal to the ambient temperature *T_b_* = *T_a_* = 20 °C [29]; we utilize the convection heat transfer coefficient of 250 W/(m^2^K) at the top surface and 150 W/(m^2^K) at the bottom surface [15] (the convection heat transfer strongly depends upon the micro-hot-plate geometry, packaging, environment, *etc*, and should be determined prior to the design [25–27]). Since the radiation heat transfer becomes more and more important at high temperatures, in order to demonstrate that the proposed model can accurately take into account radiation heat transfer throughout the device even in case of extreme operating temperatures, we consider an 800 °C temperature for the hotplate (*i.e.*, significantly higher than for most practical micro-hotplates); however, clearly, our approach is completely general.

Our model assumes perfectly circular geometry; by contrast, practical heaters require electrical contacts which disturb the circular symmetry due to both the undesirable heat generation in the electrical contacts (which may not be circular) and to the thermal conductivity of the electrical contacts (which is, generally, much higher than the thermal conductivity of the insulating membrane). However, in an earlier article, we have investigated this issue and proposed strategies for designing micro-heaters with an almost circular-temperature-symmetry [24]. Here, beside taking advantage of those strategies, we have also made the internal contacts almost circular as, obviously, this choice minimizes the undesirable heat generation (the electrical contacts become as wide as possible and, therefore, the parasitic resistance of the electrical contacts, which is responsible for undesired heating powers, become as small as possible) and also results in an almost-circular-symmetry of the thermal conductivity; both these consequences are, obviously, optimal for circular-temperature-symmetry. As an additional advantage, both the almost-circular internal-contacts and the circular plate [33] inside the heater-1 also act as heat spreading plates and, therefore, further improve the temperature uniformity within the hot-region. Clearly, the external contacts (lying in the external region) may not be arbitrarily widened as this would degrade the thermal separation between the hot-region and the bulk. Moreover, in order to improve the temperature uniformity we use three individual heaters.

**Figure 4.**
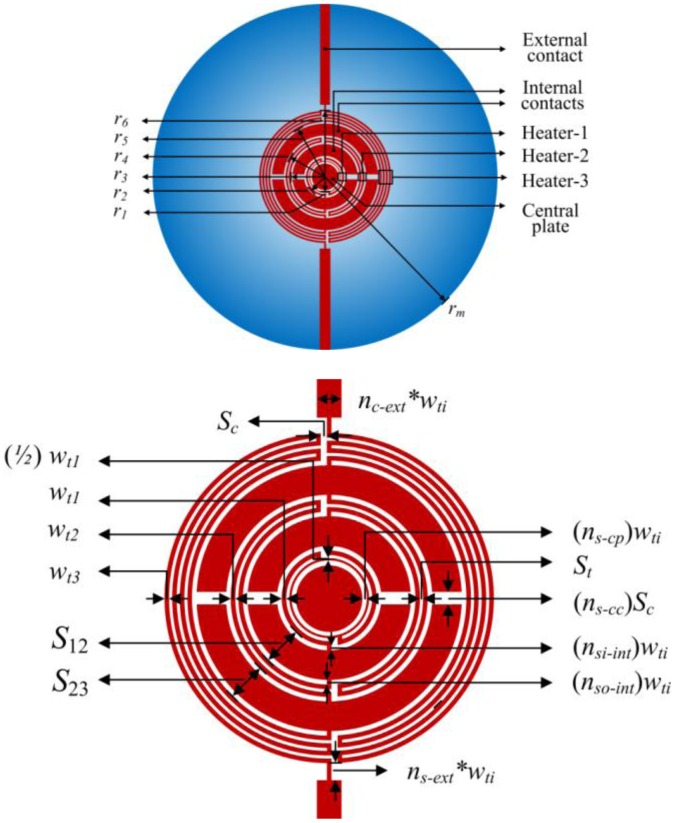
Micro-hotplate with three-heater geometry (**top**) and zoom of the central part of micro-hotplate (**bottom**) (not to scale).

The final structure is shown in Figure 4 and consists of three individual heaters where:

– *r_1_* and *r_2_* are the inner and outer radiuses of heater-1, respectively;
– *r_3_* and *r_4_* are the inner and outer radiuses of heater-2, respectively;
– *r_5_* and *r_6_* are the inner and outer radiuses of heater-3, respectively;
– *w_t1_*, *w_t2_* and *w_t3_* are the track widths of the heater-1, heater-2 and heater-3, respectively; the track width of the inner-most ring of heater-1 is half of the track width of the other parts of the heater-1;
– *r_m_* is the radius of the membrane;
– *S_t_* is the spacing between the tracks;
– *S_c_* is the length of the cuts at the extremities of the tracks length;
– *n_c-ext_* is the contact-width multiplicity factor [24] for the external contacts;
– *n_so-int_* and *n_si-int_* are the distance-to-junctions multiplicity factors for the outer and inner edges of the internal contacts, respectively;
– *n_s-ext_* is the distance-to-junctions multiplicity factor for the external contacts;
– *n_s-cc_* is the contact-to-contact-distance multiplicity-factor;
– *n_s-cp_* is the distance-to-central plate multiplicity factor.

In order to include the radiation heat transfer in the external region, our model considers the external region as consisting of 94 annular rings (*i.e.*, *N_s_*_-_*_ext_*_-_*_region_* = 94, that is the external region from *r_6_* to *r_m_* is divided into the radii of *r_7_*, *r_8_*, *r_9_*, …, *r_100_* = *r_m_*) so that the micro-hot-plate consists of total 100 regions (*J* = 100). Clearly, the annular regions must be more dense and small in proximity of the hot-region, where high temperature gradients exist, and less dense close to the bulk. In practice, a very simple and effective strategy is to define the radii of all the annular regions by taking advantage of a single FEM simulation (which will automatically determine the mesh by dividing the domain into a set of discrete elements while keeping the temperature differences between adjacent elements at reasonably small values). The micro-hotplate parameters utilized in the comparison are summarized in Table 2.

**Figure 5.**
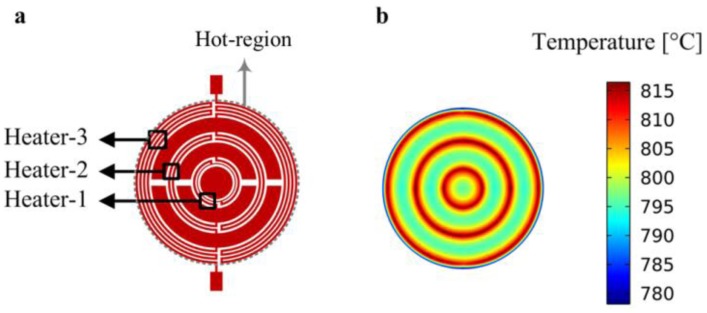
Micro-heater (**a**) and FEM results for the surface temperature distribution (°C) within the hot-region (**b**).

Figure 5 shows the FEM results for the surface temperature distribution within the hot-region, *i.e.*, micro-heater; highest temperature (*i.e.*, about 815 °C) corresponds to the heater regions (heater-1, heater-2 and heater-3), whereas we see that the temperature decreases while moving away from the heater regions; importantly, circular temperature patterns in Figure 5 demonstrate that our micro-heater design strategies provide an excellent temperature symmetry.

**Figure 6.**
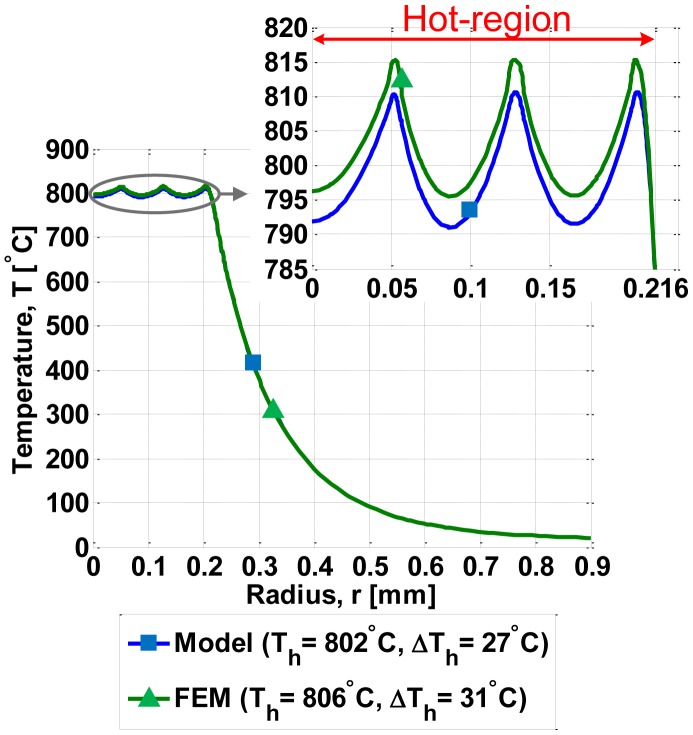
Temperature distribution in the micro-hotplate; *T_h_* is the average temperature in the hot-region and Δ*T_h_* is maximum temperature difference in the hot region; at the edge of the hot region (which extends up to 0.216 mm), there is a sharp decrease of the temperature which is responsible for the larger values of Δ*T_h_*.

**Figure 7.**
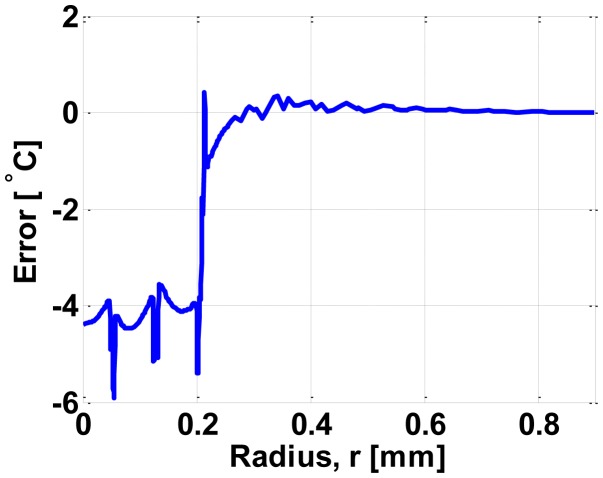
Error in the model in comparison with the FEM simulation.

Figure 6 compares our model and FEM simulations for the temperature distribution along the radius of the micro-hotplate; *T_h_* and *ΔT_h_* denote the average temperature and the maximum temperature difference (*i.e.*, difference between maximum temperature and minimum temperature) within the hot-region, respectively. In this comparison, FEM simulations, which are the most accurate available method for simulating micro-hotplates, have been used as a reference model; therefore, the error of the proposed model is simply computed as the difference between the temperature distribution predicted by the proposed model and the temperature distribution obtained by FEM simulations. Figure 7 shows the error of our model in comparison to the FEM simulations.

We stress that the almost circular internal contacts and very wide external contacts have substantially reduced the error by reducing the undesirable heat generation within the contacts (*i.e.*, by improving the circular-symmetry of the temperature distribution, which is a pre-requisite for taking full advantage of the relations in [23]). As to the computational efficiency, our model outperforms FEM simulations with an execution time around 2 s compared to more than 13 min, 20 s using FEM simulations (having 23,656 mesh elements) on an Intel(R) Core TM i3 CPU M370 @ 2.4 GHz (*i.e.*, about 400 times faster).

**Figure 8.**
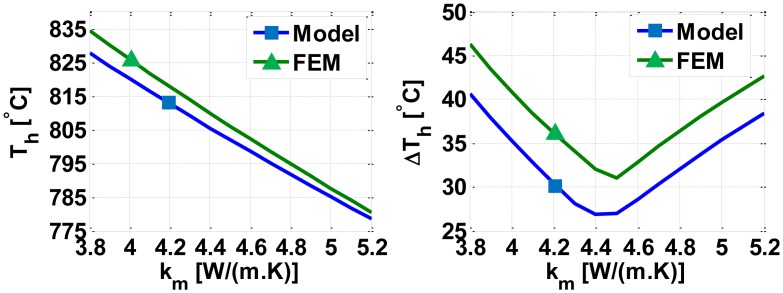
Influence of a spread in the thermal conductivity of the membrane, k_m_, on the average temperature, *T_h_* (**left**); and on the maximum temperature difference, Δ*T_h_* (**right**), in the hot region.

As another illustrative example, Figure 8 compares FEM results and our model when studying the influence of a spread of ±0.7 [W/m.K] in the thin film thermal conductivity value of 4.5 [W/m.K] [31] of the silicon nitride membrane (non-stoichiometric silicon nitride membranes can have much higher values of the thermal conductivity [34]; we, however, prefer to consider values given in [31] as this paper also contains an experimental data on the spread of the thermal conductivity; however, of course, our model can be applied to any values of the design parameters. Clearly, the spread in the thermal conductivity significantly influences both the average temperature in hot region *T_h_* and maximum temperature difference in hot region Δ*T_h_*. The average temperature in hot region *T_h_* decreases with an increase in the thermal conductivity due to correspondingly increasing conduction losses. As to the maximum temperature difference in hot region Δ*T_h_*, the minimum is found at the nominal value of the thermal conductivity, 4.5 [W/m.K], because we have designed, when considering the nominal value of the thermal conductivity, the device for a maximum temperature uniformity, *i.e.*, so that the different heaters (Heater-1, Heater-2, and Heater-3, see Figure 4) have almost the same temperature (see Figures 5 and 6). However, in presence of a spread in the thermal conductivity, the individual heaters no longer have the same temperature and, therefore, the maximum temperature difference within the hot region, *ΔT_h_*, increases (*i.e.*, the temperature uniformity is degraded). As to computational efficiency, our model performed this investigation in 9 s compared to more than 82 min using FEM simulations on an Intel(R) Core TM i3 CPU M370 @ 2.4GHz (*i.e.*, about 540 times faster).

**Figure 9.**
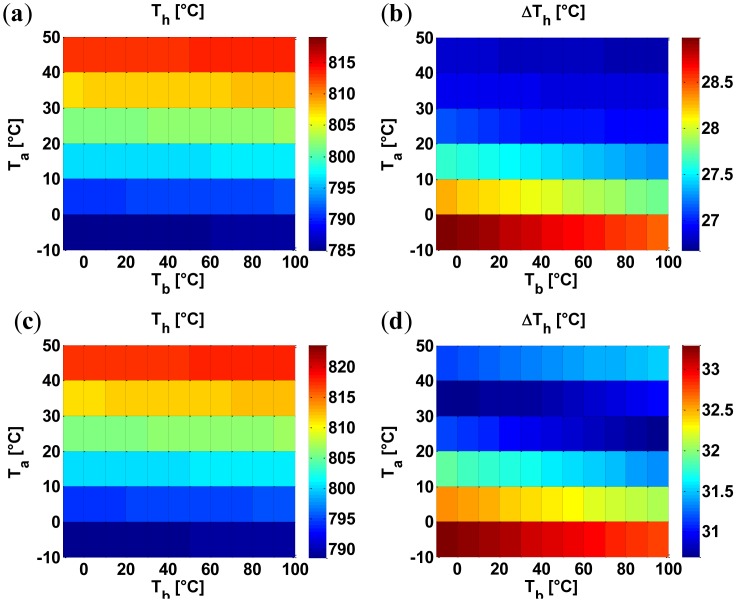
Model results (**a, b**) and FEM results (**c, d**) for the influence of a variation of the bulk temperature, *T_b_*, and ambient temperature, *T_a_*, on the average temperature in the hot region, *T_h_* (**a, c**), and maximum temperature difference in the hot region, Δ*T_h_* (**b, d**).

**Figure 10.**
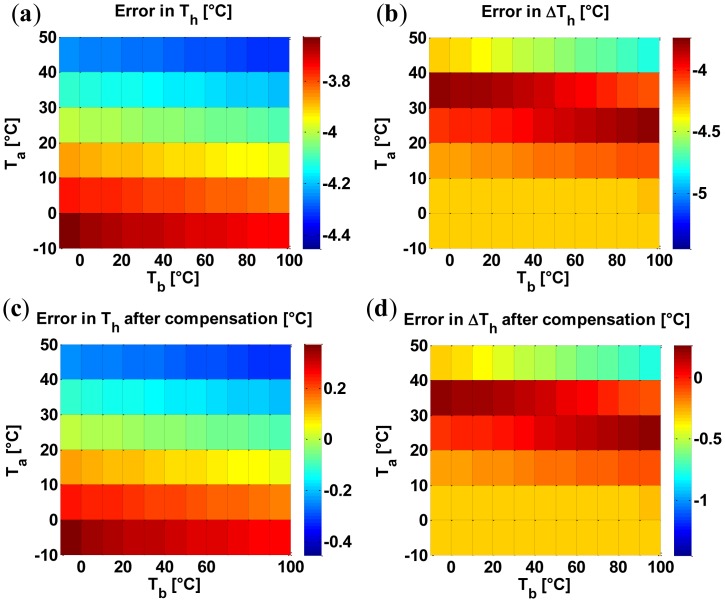
Error in model results (**a, b**) and error in model results after compensation by single FEM simulation (**c, d**) for the average temperature in hot region, *T_h_* (**a, c**) and maximum temperature difference in hot region, Δ*T_h_* (**b, d**).

As an additional example, Figure 9 presents the influence of combined variations of the ambient temperature, *T_a_*, and the bulk temperature, *T_b_*, on both the average temperature within the hot region, *T_h_*, and the maximum temperature difference within the hot region, Δ*T_h_*. In particular, we have considered a variation from –10 to 50 °C for the ambient temperature (as the gas sensors are designed to operate in this range [35]) and a variation from –10 to 100 °C for the bulk temperature (if the electronic interface [3], including the high power devices for driving the heaters, are integrated in the bulk, the bulk temperature can be substantially higher than the ambient temperature). Again, since we designed the device for a maximum temperature uniformity when considering the nominal values of the bulk and ambient temperatures, the minimum for *ΔT_h_* is found at the nominal values of the bulk and ambient temperatures. Our model performed this investigation in 45 s compared to more than 8 h using FEM simulations on an Intel(R) Core TM i3 CPU M370 @ 2.4 GHz (*i.e.*, about 640 times faster). As to the accuracy, the top row in Figure 10 shows the error between our model and FEM results for the average temperature within the hot region *T_h_* and for the maximum temperature difference within the hot region Δ*T_h_*; the error is mainly due to the undesired heat generated within the electrical contacts. Clearly, since the average temperature in the hot region does not excessively change in correspondence of the variations of both the ambient temperature and the bulk temperature, the heat generated within the electrical contacts is roughly constant and, therefore, results in an almost constant error (see the top row of Figure 10). As a result, since the error is constant, a single measurement of the error (*i.e.*, a computationally feasible single FEM simulation) is sufficient for an effective compensation. Accordingly, we have computed the error at the nominal values of the ambient and bulk temperatures (20 °C) and then subtracted it from the other errors; as evident from the bottom row in Figure 10, the proposed single-FEM-compensation reduces the errors for both the average temperature *T_h_* and the maximum temperature difference Δ*T_h_* to levels around 1 °C which, clearly, are extremely small for devices operating at about 800 °C. We stress that the single-FEM-compensation strategy is effective only for those cases which result in an almost-constant undesired heating in the electrical contacts (e.g., variations of the bulk and environment temperature) but is useless otherwise (e.g., variation of the thermal conductivity). Obviously, Figures 8, 9 and 10 (which are, to the best of our knowledge, the first reported analyses of the effects of the spread and the drift in micro-hotplates) are only illustrative of the potential of the proposed approach for the analysis, design, and optimization of membrane-type micro-hotplates.

To summarize the above discussion, the comparison with FEM simulations demonstrates that our model is almost as accurate and much faster and, therefore, represents an ideal tool for more accurate characterizations and more effective optimization of micro-hotplates.

## 4. Conclusions

Though FEM simulations are the only available tool for accurately predicting the temperature distribution in practical micro-hotplates, their computational cost is very high. As a result, micro-hotplate designers generally have no effective simulation tools for the optimization and only take into account the nominal values of the important design parameters, thus fully neglecting the effects of both spread and drift of the design parameters. In order to overcome these challenges, here, we have demonstrated an accurate and computationally efficient model for practical circular-symmetric membrane-type micro-hotplates. As to the accuracy, our model takes into account all the important design parameters such as the thermal conductivities and emissivities, electrical resistivity of the heater and (unlike any previous model) its temperature coefficient, dimensions of the micro-heater and membrane, boundary conditions, and supply voltage. In particular, by using the Taylor approximation of the radiation heat flow and simple segmentation of the membrane into annular regions so small that the Taylor approximation holds well, our approach accurately considers the radiation heat transfer in the entire micro-hotplate. For computational efficiency, our model takes advantage of simple analytical expressions for the temperature distribution in annular regions, of a matrix-based approach for imposing the relevant boundary conditions, and of an iterative procedure for estimating the average temperatures of the annular regions required for the linearization process; moreover, in case of multiple simulations, the results of the previous simulation are used as reasonable initial guesses. For validation, we have compared our model and FEM simulations in the analysis of the temperature distribution in a practical circular-symmetric three-heater micro-hotplate. Our model is two to three orders of magnitude more computationally efficient than FEM simulation (e.g., 45 s *versus* more than 8 h, *i.e.*, about 640 times faster). The residual errors, which are mainly associated to the undesired heating in the electrical contacts, are small (e.g., few degrees Celsius for an 800 °C operating temperature) and, for some analyses, almost constant so that a single (*i.e.*, computationally easy) FEM simulation may be sufficient for an effective compensation which brings the residual errors down to about 1 °C. As illustrative examples, we have reported a systematic investigation of the effects of the thermal conductivity spread and of combined variations of the ambient and bulk temperatures. Our investigations demonstrate that the proposed model, in combination with a very limited number of FEM simulations, is ideal for the systematic analysis and design of high performance micro-hotplates and may represent an extremely efficient tool for optimization of micro-hotplates.

## Figures and Tables

**Table 1.** Material properties.

**Material**	**Thermal Conductivity [W/(m·K)]**	**Resistivity [Ω·m]**	**Temperature Coefficient of Resistance [1/K]**	**Surface Emissivity**
silicon nitride	4.5 [31]	-	-	0.9
platinum	29.5 [32]	1.05 × 10^−7^	3. 927 × 10^−3^	-

**Table 2.** Micro-hotplate parameters.

**Micro-Hotplate Parameters**	**Values**
*r_m_*, *t_m_*, *t_h_*	900 μm, 1.8 μm, 0.3 μm
*r_1_*, *r_2_*	50 μm, 55 μm
*r_3_*, *r_4_*	125 μm, 132 μm
*r_5_*, *r_6_*	202 μm, 216 μm
*N_s-ext-region_*, *J*	94, 100
*w_t1_*, *w_t2_*, *w_t2_*	2 μm, 2.5 μm, 2 μm
*S_12_*, *S_23_*	70 μm, 70 μm
*S_t_*, *S_c_*	2 μm, 2 μm
*n_so-int_*, *n_si-int_*, *n_s-cc_*, *n_c-ext_*, *n_s-ext_*, *n_s-cp_*	1, 1, 4, 12, 12, 1
Supply voltage, *V_s_*	27.2 V
